# ASMase Activation in Ultrasound-Stimulated Radiation Enhancement Using MRI-Guided Focused Ultrasound

**DOI:** 10.3390/cells14201618

**Published:** 2025-10-17

**Authors:** Tera N. Petchiny, Deepa Sharma, Anoja Giles, Kai Xuan Leong, Wenyi Yang, Lakshmanan Sannachi, David Alberico, Gregory J. Czarnota

**Affiliations:** 1 Physical Sciences, Sunnybrook Research Institute, Toronto, ON M4N 3M5, Canada; tera.petchiny@mail.utoronto.ca (T.N.P.);; 2 Department of Laboratory Medicine and Pathobiology, University of Toronto, Toronto, ON M4N 3M5, Canada; 3 Department of Medical Biophysics and Radiation Oncology, Temerty Faculty of Medicine, University of Toronto, Toronto, ON M4N 3M5, Canada; 4 Department of Radiation Oncology, Sunnybrook Health Sciences Centre, Toronto, ON M4N 3M5, Canada

**Keywords:** focused ultrasound, microbubbles, radiation enhancement, MRI-guided focused ultrasound, ASMase, histology, cellular structure

## Abstract

Focused ultrasound-stimulated microbubble (MB + FUS) therapy is a promising radiation enhancement strategy, utilizing vascular disruption to enhance radiation efficacy. However, its mechanistic effects in large tumour volumes and clinical translatability remain insufficiently characterized. This study evaluates the synergistic impact of MB + FUS combined with radiation therapy (XRT) in a large-scale, immunosuppressed rabbit xenograft model using a clinically adaptable, MRI-guided 6144-element focused ultrasound (MRgFUS) system. Tumours were treated with MB + FUS, XRT, or both, with real-time image-guided MB activation and evaluation of treatment effects on vascular integrity, proliferation, and cellular stress responses. Assessments included Power Doppler ultrasound, histology, and immunohistochemistry targeting TUNEL, ASMase, Ki-67, Factor VIII, HIF-1α, and VEGF. Combination therapy induced significant vascular collapse, reduced perfusion, and decreased Factor VIII expression, alongside increased nuclear condensation, TUNEL positivity, and ASMase expression, consistent with ASMase-mediated endothelial apoptosis and vascular disruption. Upregulation of HIF-1α and VEGF indicated hypoxia-driven angiogenic signalling, while Ki-67 suppression reflected sustained tumour growth inhibition. Although immune responses were limited by host immunosuppression, the larger tumour burden provided clinically relevant constraints. The MRgFUS platform enabled precise and reproducible MB activation, reinforcing MB + FUS as a potent radio-enhancement modality. These findings support the continued development of MB + FUS toward clinical translation and highlight its potential as a complementary strategy to radiation therapy.

## 1. Introduction

Radiation therapy (XRT) remains a cornerstone of contemporary oncologic care, with over half of cancer patients receiving XRT as a primary, adjuvant, or palliative intervention. Its therapeutic efficacy arises from a combination of direct and indirect mechanisms, including direct DNA damage and indirect generation of reactive oxygen species (ROS) via radiolysis of intracellular water [1,2,3,4,5]. Ionizing radiation directly induces DNA single-strand (SSBs) and double-strand breaks (DSBs), effectively overwhelming the cell’s repair machinery and initiating programmed cell death [1,2,3,5,6]. Simultaneously, the radiolysis of water molecules generates ROS, such as hydroxyl radicals (•OH), superoxide (O_2_^−^), and hydrogen peroxide (H_2_O_2_), which collectively inflict oxidative damage to DNA, proteins, and lipids, thus amplifying cellular stress responses [2,6,7]. Tumour cells, which often have impaired oxidative stress defences, are particularly vulnerable to ROS-induced injury [4,6,7,8,9]. However, this oxidative mechanism also presents a paradox as the hypoxic and antioxidant-rich tumour microenvironment can diminish ROS efficacy and contribute to radioresistance [3,4,8,9,10,11]. Moreover, because ROS are non-specific and diffusible, excessive production can also harm adjacent healthy tissue, necessitating careful modulation of XRT dose and delivery [2,8].

Despite its wide clinical applications, XRT efficacy is frequently constrained by tumour hypoxia, radioresistance, and off-target toxicity [8,10,11]. Hypoxic tumour microenvironments suppress ROS generation and impede the oxygen fixation reaction necessary for stabilizing DNA radicals, thereby diminishing radiation-induced cytotoxicity [11,12]. Additionally, high-dose radiation is associated with adverse effects on adjacent normal tissues, including gastrointestinal, neurological, and hematopoietic systems, emphasizing the need for strategies that enhance radiosensitization while preserving normal tissue integrity [3,10,11,12].

Microbubbles (MBs) stimulated by focused ultrasound (FUS) have emerged as a promising radiation-enhancing modality capable of overcoming several of these limitations [13,14]. MBs are composed of gas-filled spheres, ranging between 1–10 µm in diameter, encapsulated in protective lipid, protein, or polymer shells [13,14,15,16]. Initially used as contrast agents in biomedical imaging, MBs enhance ultrasound imaging by increasing acoustic backscatter within vascular regions, facilitating real-time visualization of tissue perfusion and blood flow [13,14,15,17,18,19]. Over the past decade, MBs have been repurposed from passive imaging agents to multifunctional therapeutic carriers. Advances in MB design, including control over size, shell structure, and surface chemistry, now enable site-specific targeting, controlled drug delivery, and activation triggered by external stimuli [14,17,20,21]. When combined with FUS, MBs are acoustically stimulated (ultrasound-stimulated microbubbles; USMB) for a variety of therapeutic functions. In this context, MBs undergo either stable (rhythmic oscillation) or inertial cavitation (violent collapse), producing localized mechanical stresses on surrounding tissues and cellular structures [16,20,21,22,23]. These mechanical effects, which include fluid microstreaming, shear stress, and acoustic radiation forces, have emerged as powerful tools for enhancing therapeutic efficacy [21,22,24]. The mechanical stress generated by USMB can increase membrane permeability, disrupt endothelial junctions, and open physical barriers, such as the blood–brain barrier (BBB), thereby enabling the localized delivery of therapeutic agents that would otherwise be inaccessible [7,14,18,21,23,24,25,26].

USMB has been extensively investigated for BBB disruption, drug delivery, and tumour ablation through means of inertial cavitation and vascular disruption [14,22,23,26,27]. Inertial cavitation results in violent bubble collapse, generating intense mechanical forces that fragment cellular membranes and damage the surrounding vasculature, promoting tumour ischemia [14,16,22,27,28]. Furthermore, the vascular collapse induced by USMB has been shown to reduce tumour perfusion, induce endothelial apoptosis, and reinforce anti-tumour effects, making USMB a promising strategy for non-thermal tumour ablation [11,16,23,26,27,28].

In contrast to conventional XRT, which primarily targets the tumour parenchyma, USMB selectively affects the tumour vasculature, a compartment that is structurally less heterogeneous and more mechanically responsive [27]. USMBs exert their effects by inducing mechanical disruption of the vascular endothelium, subsequently triggering activation of the acid sphingomyelinase (ASMase)/ceramide signalling pathway [16,27,29,30]. This process is driven by inertial cavitation, defined as the unstable oscillation of MB under specific ultrasound frequencies and pressures, culminating in their eventual collapse [16,27,31,32]. The resulting mechanical forces from MB collapse exert intense localized stress that physically disrupts the endothelial lining at the target site [16,19,27,31]. These biomechanical perturbations initiate translocation of ASMase to the outer plasma membrane, where it catalyzes the hydrolysis of sphingomyelin into ceramide [16,22,29,30,33]. Ceramide, a pro-apoptotic sphingolipid, accumulates in the membrane and forms lipid raft microdomains that cluster death receptors (e.g., Fas/CD95, TNF-R1, and DR5), leading to the assembly of the death-inducing signalling complex (DISC) and promoting initiation of the extrinsic apoptotic cascade through caspase-8 activation [6,30]. Simultaneously, ceramide facilitates mitochondrial outer membrane permeabilization (MOMP), either via membrane pore formation directly or through the recruitment of pro-apoptotic proteins (Bax and Bak) [6,32]. This leads to the release of cytochrome c, activation of caspase-9, and the execution of apoptosis through caspase-3, also known as the intrinsic pathway [6,32]. Collectively, ceramide functions as a bioactive mediator of endothelial apoptosis, vascular collapse, and enhanced tumour radiosensitization [28,29].

Previous studies, such as those by Afrin et al. [30] and Leong et al. [33], have confirmed the central role of ASMase in mediating USMB-enhanced cell death. In murine models with ASMase knockout or inhibition, the therapeutic benefits of USMB were shown to be negated, confirming the ASMase/ceramide pathway as an essential aspect of USMB’s radio-enhancement potential [16,29]. However, these investigations have largely relied on ceramide quantification as a surrogate for pathway activation. In this study, we employ a commercially available anti-ASMase antibody for in situ detection of ASMase activity, marking, to our knowledge, the first immunohistochemical application of this antibody in the context of USMB and XRT. This approach enables mechanistically specific and spatially resolved analysis of treatment effects.

In addition to this molecular characterization, the current work addresses a key translational gap in the field. Most prior preclinical studies have been confined to small tumour models in rodents, which inadequately recapitulate the spatial complexity, perfusion heterogeneity, and hypoxic microenvironments of human tumours [19,22,27,33,34,35]. To address this limitation, the work here developed a large-scale in vivo model. In order to experimentally validate these mechanistic insights and translate them to clinical relevance, the aim of the study here was to characterize how USMB and XRT influence the vasculature of large tumours in vivo. Subcutaneous prostate xenografts were established in immunosuppressed rabbits to generate large, heterogeneous tumours more representative of clinical complexity. Four treatment groups were evaluated: MB + FUS (representing USMB), XRT alone (8 Gy), combined MB + FUS + XRT (8 Gy), and an untreated control. Acute vascular response was monitored using Power Doppler (PD) ultrasound to measure blood flow before and after treatment. After treatments, tumours were excised and analyzed using histology and immunohistochemistry to quantify cell proliferation, vascular injury, apoptosis, ASMase expression, hypoxia, and angiogenesis.

## 2. Materials and Methods

### 2.1. Cell Culture

A human-derived prostatic adenocarcinoma cell line, known as PC-3 (ATCC CRL-1435, Manassas, VA, USA), was used for this experiment to generate a rabbit xenograft model. PC-3 cells were cultured in Roswell Park Memorial Institute (RPMI) 1640 Medium with L-Glutamine & Sodium Bicarbonate, 1× (Wisent Inc., Quebec, Canada. Catalogue No. 350-000 CL), supplemented with 10% (*v*/*v*) characterized Fetal Bovine Serum (FBS; Hyclone, Logan, UT. Catalogue No. SH3039603) and 1% (*v*/*v*) Penicillin-Streptomycin (Gibco, Grand Island, NY, USA. Catalogue No. 15140122). Cells were seeded in 175 cm^2^ flasks and incubated at 37 °C with 5% CO2. The media was replaced every 3–4 days, or when the pH decreased below 7 (pH ≤ 6.8), which was indicated by a colour change from red to yellow/brown. When cells became confluent, typically 7 days after seeding, cells were split using 4 mL of Trypsin-EDTA (0.05%; Gibco, Grand Island, NY, USA. Catalogue No. 25300062). Cells were passaged 6-8 times before preparation for injection. Prior to inoculation, cells were counted using a hemocytometer to establish a concentration of 10 million cells (1 × 10^7^)/500 µL per injection. During cell preparation, cells were collected using standard centrifugation and the pellet resuspended in 1 mL of Phosphate-Buffered Saline 1× (PBS) with Calcium & Magnesium (Wisent Inc., Quebec, Canada. Catalogue No. 311-011 CL) and subsequently diluted to a 1:10 ratio with PBS. A 10 µL aliquot of the diluted solution was deposited on the hemocytometer and counted according to the recommended counting procedure. The total cell count for 1 mL was calculated using the following equation:

Total Cell Count (viable cells/mL) = Average Cell Count × 100 (Dilution) × 10^4^ (Hemocytometer Calibration) (1)

Once cells were counted, 10 million cells (1 × 10^7^) suspended in PBS, approximately two confluent 175 cm^2^ flasks per rabbit, were prepared for tumour cell inoculation.

### 2.2. Animal Management

All in vivo experiments were approved by the Sunnybrook Research Institute (SRI) Animal Care Committee (ACC; AUP 25-939). Animal handling adhered to guidelines from the Canadian Council on Animal Care (CCAC). New Zealand White male rabbits (Charles River Laboratories, Quebec, Canada) were received by the SRI ACC staff, and their weight and general health status were documented upon arrival. The rabbits were stored in a HEPA-filtered, positive-pressure clean room, in independent housing units equipped with recommended enrichment items. The rabbits were acclimatized to the new environment for two weeks from the arrival date, with daily interactions from pre-clinical staff before the start of the experiment. The rabbits were monitored and weighed daily until reaching a minimum of 2 kg before commencing with immunosuppression. A total of 20 rabbits were used, randomly assigned into four groups (*n* = 5 per group): Control, XRT, MB + FUS, and MB + FUS + XRT.

### 2.3. Immunosuppression

Once rabbits reached an appropriate weight (≥2 kg), rabbits were injected with Cyclosporine A (CsA; Sandimmune, Novartis Pharmaceuticals Canada Inc., Montreal, QUE, Canada. DIN No. 00593257) to suppress the immune system and prevent tumour cell rejection. A dose of 15mg/kg of CsA diluted in 4 mL of saline (0.1%) was administered intramuscularly 24 h before tumour cell inoculation and then injected daily for the duration of the experiment, or until the rabbit was euthanized. The CsA and saline were mixed thoroughly and injected into the dorsal region of the rabbit using a 25G needle. Each day, the injection site was rotated between four dorsal quadrants to allow for site recovery and reduce the chance of injury. After 24 h, PC-3 cells were subcutaneously injected into the upper aspect of the right hind leg of the rabbits. Tumours were grown for approximately 4 weeks, or until the tumour was 2 cm in size, before commencing imaging and treatments.

### 2.4. Ultrasound Imaging

Rabbits were anesthetized using 3–5% isoflurane (Fresenius Kabi, Toronto, ON, Canada. Catalogue No. 02237518) in oxygen administered by nosecone using a precision vaporizer. The hair surrounding the tumour was removed using a depilatory cream (Nair hair removal cream) before imaging.

Rabbits were placed on a heating pad to maintain consistent body temperature throughout the scanning procedure. The tumour-bearing leg was completely submerged in a custom-built water bath (warmed) and mounted on a vibration-damping platform to stabilize the setup and prevent motion artifacts. Ultrasound imaging was completed 24 h before (Pre) and 24 h after (Post) treatments.

For all pre-clinical imaging, the Vevo770 (VisualSonics, FUJIFILM, Mississauga, ON, Canada) system was utilized with a 710B probe, consisting of a 25 MHz central frequency, an axial resolution of 70 µm, a lateral resolution of 140 µm, a depth of 2.7 mm, and a focal length of 15 mm. The Vevo770 ultrasound machine was used to collect B-mode, radiofrequency (RF), and 3D Power Doppler (PD) data. PD scanning was performed at a medium velocity with the following parameters: gain of 20 dB, step size of 0.2 mm, wall filter of 2.5 mm/s, and a scan speed of 2.0 mm/s.

### 2.5. Microbubble Preparation and Administration

Definity MB (Lantheus Medical Imaging, North Billerica, MA, USA; DIN No. 0.2243173), composed of perfluoropropane gas within a phospholipid shell, were used in all USMB experiments. Definity MB has a mean diameter of 1.1–3.3 µm, with 98% of bubbles less than (<) 10 µm in size, as characterized by the manufacturer and consistent with prior studies [13]. Vials were brought to room temperature and activated via agitation using a Vialmix (Lantheus Medical Imaging) for 45 s. A single vial (1.5 mL at 1.2 × 10^10^ MB/mL) was prepared for each rabbit and diluted with 4 mL of sterile saline, yielding a final volume of 5.5 mL and a working concentration of 3.27 × 10^9^ MB/mL. After activation and dilution, the entire 5.5 mL solution was administered to a 2 kg rabbit intravenously. This corresponds to a total dose of ~9 × 10^9^ MB/kg, approximating 0.75 mL/kg of Definity MB in relation to the original vial content. This established a high concentration of MB in circulation during FUS therapy. The solution was administered intravenously via an ear-vein catheter using a power injector over 5–6 min. This infusion rate (~1 mL/min) was selected to maintain stable MB concentrations in circulation during the 5-min FUS treatment, minimizing hemodynamic stress and ensuring sufficient overlap between MB in circulation and ultrasound exposure. Based on previous pharmacokinetic studies, Definity MB reaches a steady-state concentration within 30–60 s after injection, with a circulation half-life of ~1.3 min [25,26].

### 2.6. Focused Ultrasound Therapy

A 6144-element FUS system (Arrayus Technologies, Burlington, ON, Canada) was integrated with a 3T MRI scanner (Achieva, Philips Healthcare, Best, The Netherlands) to enable precise treatment planning and anatomical targeting along the tumour region. FUS therapy was delivered using a peak negative pressure of 570 kPa and a center frequency of 500 kHz. Sonications were applied using a pulse duration of 50 milliseconds at a 25% duty cycle, resulting in periodic ultrasound exposure tailored to promote mechanical disruption while avoiding thermal damage. The focal volume of the array transducer was 2.3 mm (transaxial) × 2.2 mm (lateral) × 7.2 mm (axial), as defined by the −6 dB acoustic intensity. To cover the entire tumour volume (~2 cm diameter), approximately 100 focal targets were used. The total treatment duration was approximately 5 min. FUS therapy commenced immediately following the start of MB infusion. No passive cavitation detection was performed during treatment. These acoustic parameters were selected because prior studies have demonstrated that they promote efficient MB inertial cavitation and associated vascular disruption, minimizing thermal tissue damage, while maximizing targeted mechanical stress [16,26,31,36].

### 2.7. Radiation Therapy

**Figure 1:**
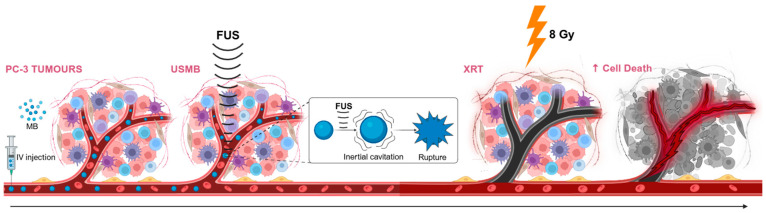
Schematic of the MB + FUS + XRT combined treatment mechanisms. Microbubbles (MB) are administered via intravenous (IV) injection and circulate systemically. Focused ultrasound (FUS) is then applied to the tumour region, inducing inertial cavitation of MBs within the tumour microvasculature (USMB), which mechanically disrupts tumour vasculature. This mechanical effect enhances vascular permeability, priming the tumour for subsequent exposure to radiation therapy (XRT). A single dose of 8 Gy is administered after FUS treatment. The combined treatment leads to increased vascular and DNA damage, resulting in enhanced tumour cell death, as indicated by the darker regions in the final panel.

Immediately following MB + FUS treatment, the rabbit was transferred for XRT. Under continued anesthesia, the animal was positioned in the Faxitron X-ray cabinet irradiator (Faxitron, Tucson, AZ, USA) with a custom lead shield covering the head, torso and non-targeted limbs, leaving only the tumour region (~4–5 cm^2^) exposed to minimize off-target radiation. The rabbit received 200 cGy/min for 4 min, resulting in a total dose of 8 Gy delivered to the targeted area. The rabbit was treated with a single high dose of 8 Gy. After treatment, the animal was monitored during recovery from anesthesia and returned to its housing unit. See Figure 1 for a schematic overview of the combined treatment (MB + FUS + XRT) mechanisms.

### 2.8. Post-Treatment Imaging and Tissue Harvesting

Twenty-four hours after treatment (MB + FUS, XRT, or MB + FUS + XRT), post-treatment ultrasound imaging was performed to assess tumour response. The analysis of the PD data was performed using an in-house program in MATLAB (vR2014a, MathWorks, Natick, MA, USA) designed to calculate changes in blood perfusion across the tumour volume, referred to as the Vascular Index (VI). To isolate the tumour, regions of interest (ROI) were manually defined based on the volumetric B-mode scans. The Doppler signal, indicated by grouped coloured pixels, observed in each ROI was measured and averaged to calculate the relative VI for the entire tumour. The changes in VI were calculated using the following formula:

Vascular Index (VI) = (VI_Post_/VI_Pre_) × 100 (2)

Following imaging, the rabbit was euthanized, and the tumour was surgically excised and sectioned axially into multiple slices. Tissue sections were placed into cassettes for subsequent processing and embedding. Half of the tumour tissue was fixed in 10% neutral buffered formalin (NBF; Thermo Fisher Scientific, Waltham, MA, USA; Catalogue No. SF934) for histological analysis. The remaining tissue was embedded in Optimal Cutting Temperature (OCT) Compound (Scigen, Paramount, CA, USA; Catalogue No. 23-730-625), flash-frozen in liquid nitrogen (LN), and stored at −80 °C for future analysis. After 48–72 h, the formalin-fixed samples were transferred to 70% ethanol, embedded in paraffin, and submitted for histological processing. Only the formalin-fixed, paraffin-embedded (FFPE) sections were used for the analyses reported in this study.

### 2.9. Histology and Immunohistochemistry

All histology and immunohistochemistry staining were outsourced to the Pathology Research Program (PRP) Laboratory at Toronto General Hospital, part of the University Health Network (UHN) research organization (Toronto, ON, Canada).

Formalin-fixed tissue was embedded in paraffin blocks and sectioned into 5 μm slices. These sections were mounted on microscope slides for histological and immunohistochemical analyses. Hematoxylin and eosin (H&E) staining was performed to evaluate structural and morphological changes within the tumour tissue. Apoptotic cell death was assessed using the Terminal deoxynucleotidyl transferase dUTP nick end labelling (TUNEL) assay, which detects DNA fragmentation by labelling the exposed 3′-hydroxyl termini using terminal deoxynucleotidyl transferase (TdT) and labelled deoxyuridine triphosphates (dUTPs).

To examine molecular pathways associated with cell death, immunohistochemical staining with an ASMase was employed to assess cellular stress and initiation of the ASMase-mediated cell death pathway. Cellular proliferation was evaluated using Ki-67 immunostaining. Vascular endothelial cells were identified via Factor VIII staining, which was also used to assess microvessel density within tumour tissue. The presence of hypoxia in the tumour microenvironment was determined by immunostaining for Hypoxia-Inducible Factor 1 alpha (HIF-1α). Angiogenesis was evaluated using Vascular Endothelial Growth Factor (VEGF) staining.

### 2.10. Whole Slide Imaging and Analysis

Whole slide images (WSI) were acquired using the TissueScope LE scanner (Huron Digital Pathology, St. Jacobs, ON, Canada) with brightfield illumination at 20X magnification, resulting in a resolution of 0.5 µm/pixel.

Quantification of staining, excluding Factor VIII, was performed using QuPath (v0.5.1, University of Edinburgh, Edinburgh, UK), an open-source bioimage analysis software [37]. Regions of interest (ROIs) were manually annotated, and cell detection was conducted using default or marker-specific optimized parameters. For H&E-stained sections, nuclei were detected using the StarDist plugin integrated within QuPath, which applies a deep-learning model for accurate nucleus segmentation [37]. For immunohistochemical stains, positive cell counts, staining intensity, and percentage area stained were quantified. The Ki-67 proliferation index was calculated as the percentage of Ki-67-positive nuclei within each ROI. For vascular and hypoxia markers, positively stained areas of the tumour were annotated and quantified. Colour deconvolution was applied to separate chromogenic stains (e.g., hematoxylin, DAB) to enhance specificity and accuracy in marker quantification.

Factor VIII was analyzed independently from QuPath. For each tumour section, 20 ROIs were manually selected and uniformly distributed across the tissue. These ROIs corresponded to the field of view of the Olympus DP73 camera on an Olympus BX53 upright microscope at 10× magnification. Based on scale bar calibration, each ROI measured approximately 922.3 µm × 670.5 µm (∼618,900 µm^2^). These ROIs were imaged individually, and positively stained vessels were manually counted in each image. Counts were averaged across the ROIs to obtain a vessel density value for each slice. Vessel counts for each specimen were calculated by averaging values from all associated slices.

Statistical analysis was conducted using GraphPad Prism 10 (v10.5.0) for Windows (Boston, MA, USA). One-way ANOVAs were followed by appropriate multiple comparisons tests, depending on variance homogeneity, to assess differences between groups.

## 3. Results

### 3.1. Histology and Immunohistochemistry

#### 3.1.1. H&E

**Figure 2:**
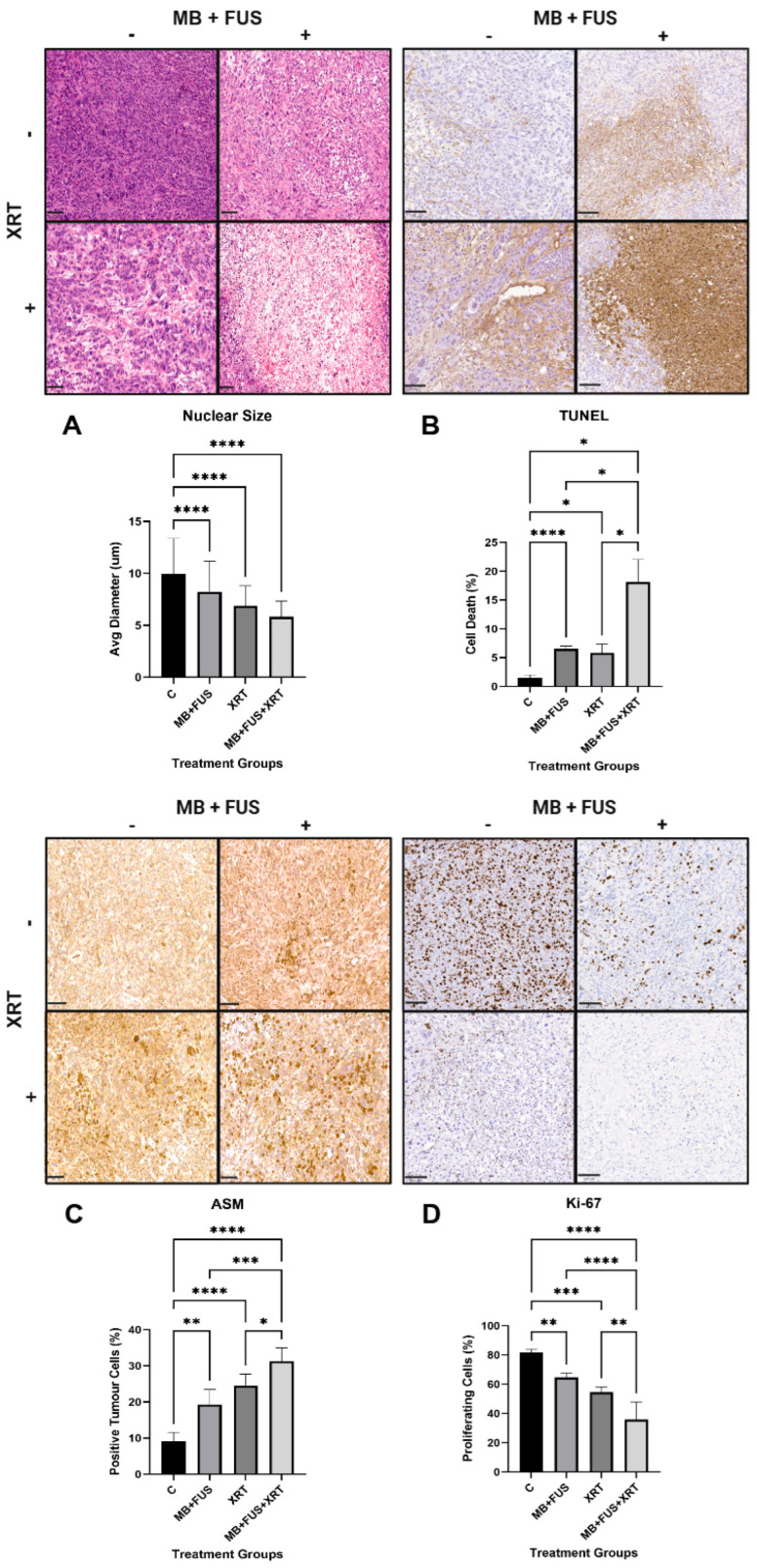
Histological and immunohistochemical analysis of tumour response. Top Rows: Representative images show (**A**) H&E staining, (**B**) TUNEL assay, (**C**) ASMase, and (**D**) Ki-67 immunostaining for tumours across four treatment groups: untreated controls (C), microbubble-enhanced focused ultrasound (MB + FUS), single-dose radiation therapy (XRT, 8 Gy), and their combination (MB + FUS + XRT). Scale bar: 100 µm. Bottom Rows: Quantification of (**A**) nuclear size, (**B**) TUNEL-positive (apoptotic) cells, (**C**) ASMase-positive tumour cells, and (**D**) Ki-67-positive proliferating cells across treatment groups. MB + FUS + XRT combination treatment resulted in significant nuclear condensation, increased cell death, elevated ASMase expression, and reduced proliferation compared to controls and monotherapies. Data are presented as mean ± SD. *p*-values ranging from *p* < 0.05 (*), *p* < 0.01 (**), *p* < 0.001 (***), *p* < 0.0001 (****).

Quantitative nuclear morphometry was used to assess nuclear condensation, reflected as reduced nuclear size, which is a hallmark of apoptosis, enabling direct comparison across treatment groups. Nuclear size, measured by average nuclear diameter, was the largest in the control group (mean ± standard deviation [SD]: 10 µm ± 3 µm, 95% CI: 10 to 10), illustrated in Figure 2. While the other treatment groups showed a steady decline in nuclear size across the MB + FUS (8 µm ± 3 µm, 95% CI: 8 to 8), XRT (7 µm ± 2 µm, 95% CI: 7 to 7), and MB + FUS + XRT (6 µm ± 2 µm, 95% CI: 6 to 6) cohorts. A Welch’s ANOVA revealed significant differences in nuclear diameter among treatment groups (W = 396.2, DFn = 3.000, DFd = 1401, *p* < 0.0001). Tests for homogeneity of variances, including the Brown-Forsythe test (F* = 331.6, DFn = 3.000, DFd = 2606, *p* < 0.0001) and Bartlett’s test (χ^2^ = 433.4, *p* < 0.0001), confirmed a significant violation of the equal variance assumption. As a result, the Games-Howell post hoc test, which does not assume equal variances and controls for multiple comparisons, was employed. All treatment groups showed a significant reduction in nuclear size compared to the control (*p* < 0.0001), as shown in Figure 2A. The MB + FUS + XRT treatment exhibited the most significant reduction, consistent with cell death, demonstrating a 40% decrease in nuclear size relative to the control (mean difference [MD] = 4, 95% CI: 4 to 5, *p* < 0.0001), shown in Figure 2A. Pairwise comparisons also revealed significant differences among treatments: XRT vs. MB + FUS (MD = −1, 95% CI: −2 to −1, *p* < 0.0001), XRT vs. MB + FUS + XRT (MD = 1, [95% CI: 1 to 1, *p* < 0.0001), and MB + FUS vs. MB + FUS + XRT (MD = 2, 95% CI: 2 to 3, *p* < 0.0001).

#### 3.1.2. TUNEL

TUNEL staining, which detects DNA fragmentation during late-stage apoptosis, was used to assess cell death and treatment response following MB + FUS + XRT. The mean percentages of cell death, as measured by TUNEL-positive staining, were as follows: Control (2% ± 0.4%, 95% CI: 1 to 2); XRT (6% ± 2%, 95% CI: 3 to 8); MB + FUS (7% ± 0.5%, 95% CI: 6 to 7); and MB + FUS + XRT (18% ± 4%, 95% CI: 12 to 24). One-way ANOVA revealed a significant difference in cell death across treatment groups (F = 51.20, *p* < 0.0001, R^2^ = 0.9220). Due to violation of homogeneity of variance (Brown-Forsythe test: F = 5.88, *p* = 0.0092; Bartlett’s test: χ^2^ = 14.86, *p* = 0.0019), Welch’s ANOVA was performed, confirming significant differences among group means (W = 70.81, *p* < 0.0001). Post hoc analysis using Games-Howell’s test showed that MB + FUS (*p* < 0.0001), XRT (*p* = 0.0304), and MB + FUS + XRT (*p* = 0.0100) significantly increased the percentage of cell death compared to the control, shown in Figure 2B. Furthermore, MB + FUS + XRT resulted in significantly more cell death than either XRT (MD = 12, 95% CI: 4 to 21, *p* = 0.0153) and MB + FUS (MD = 12, 95% CI: 2 to 21, *p* = 0.0266), respectively. No significant difference was found between XRT and MB + FUS (*p* = 0.8398).

#### 3.1.3. ASMase

ASMase was evaluated as a marker of cellular stress and tumour response, as it plays a key role in ASMase-mediated endothelial apoptosis and vascular disruption induced by both USMB and XRT. Quantitative analysis of ASMase staining, reflecting activation of the ASMase/ceramide pathway, showed significant variation across treatment groups. The control group exhibited the lowest mean value at 9% ± 3% (95% CI: 6 to 12), as shown in Figure 2C. The MB + FUS group demonstrated a notable elevation with a mean of 19% ± 4% (95% CI: 14 to 24). The XRT group showed a higher mean of 25% ± 3% (95% CI: 20 to 30). The highest response was observed in the MB + FUS + XRT group, with a mean of 31% ± 4% (95% CI: 27 to 36), see Figure 2C. One-way ANOVA confirmed a significant treatment effect (F = 37.36, *p* < 0.0001), with 88.2% of total variance explained (R^2^ = 0.8820). Assumptions of homogeneity of variance were met, with non-significant results from both Brown-Forsythe (*p* = 0.8630) and Bartlett’s (*p* = 0.8247) tests. Post hoc analysis using Tukey’s multiple comparisons test revealed that all treatments significantly increased ASMase expression relative to the control: Control vs. MB + FUS (MD = –10, 95% CI: −16 to −4, *p* = 0.0013), Control vs. XRT (MD = −15, 95% CI: −22 to −9, *p* < 0.0001), and Control vs. MB + FUS + XRT (MD = −22, 95% CI: −28 to −16, *p* < 0.0001). While MB + FUS and XRT did not differ significantly from each other (MD = −5, 95% CI: −12 to 1, *p* = 0.1484), the combination MB + FUS + XRT yielded significantly higher values than either MB + FUS (MD = −12, 95% CI: −18 to −6, *p* = 0.0003) or XRT alone (MD = −7, 95% CI: −13 to −0.2, *p* = 0.0438).

#### 3.1.4. Ki-67

Ki-67 analysis was used to assess cellular proliferation and treatment response, observing whether MB + FUS + XRT effectively suppresses tumour growth. The mean percentage of proliferating cells, evaluated by Ki-67 staining, indicated the highest degree of proliferating cells in the control group (82% ± 2%, 95% CI: 79 to 84), with a progressive decline in proliferation observed in the MB + FUS (65% ± 3%, 95% CI: 61 to 68, XRT (55% ± 4%, 95% CI: 49 to 60), and MB + FUS + XRT (36% ± 12%, 95% CI: 21 to 51) treatment groups, shown in Figure 2D. A one-way ANOVA revealed a highly significant difference in Ki-67 expression among the four treatment groups (F = 41.40, *p* < 0.0001), with a large effect size (R^2^ = 0.8923), indicating that approximately 89.2% of the total variance in Ki-67 expression was attributable to treatment effects. Despite Bartlett’s test suggesting heterogeneity of variances (*p* = 0.0042), the Brown-Forsythe test was non-significant (*p* = 0.6113), supporting the robustness of the ANOVA results. Tukey’s post hoc comparisons showed the MB + FUS + XRT group significantly differed from the Control group (MD = −46, 95% CI: −52 to −40), the XRT group (MD = −19, 95% CI: −28 to −9), and the MB + FUS group (MD = −29, 95% CI: −38 to −20). The Control group also differed significantly from the XRT group (MD = 27, 95% CI: 18 to 37, *p* < 0.0001) and the MB + FUS group (MD = 17, 95% CI: 8 to 26, *p* = 0.0053), while no significant difference emerged between the XRT and MB + FUS groups.

#### 3.1.5. Factor VIII

**Figure 3:**
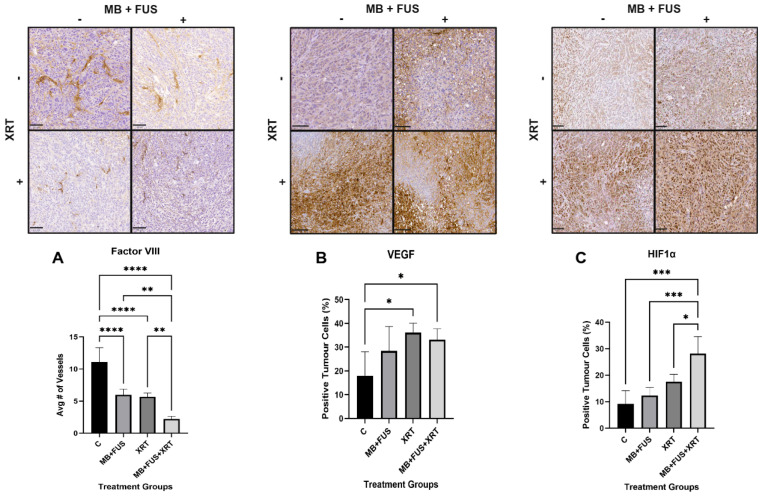
Immunohistochemical analysis of vascular and hypoxic tumour response. Top Row: Representative images show staining for (**A**) Factor VIII (vascular marker), (**B**) VEGF (angiogenesis factor), and (**C**) HIF1α (hypoxia marker) in tumours from control (**C**), microbubble-enhanced focused ultrasound (MB + FUS), single-dose radiation therapy (XRT, 8 Gy), and combined MB + FUS + XRT (single-dose, 8 Gy) treatment groups. Scale bar: 100 µm. Bottom Row: Quantification of (**A**) average number of Factor VIII-positive vessels per region of interest (ROI), (**B**) percentage of VEGF-positive tumour cells, and (**C**) percentage of HIF1α-positive tumour cells demonstrates that MB + FUS + XRT significantly reduces vascularization while elevating hypoxia markers. Images are representative of each group, with a scale bar of 100 µm. Data are presented as mean ± SD. *P*-values ranging from *p* < 0.05 (*), *p* < 0.01 (**), *p* < 0.001 (***), *p* < 0.0001 (****).

Factor VIII staining, an endothelial marker, was used to assess vascular integrity and microvessel density (MVD). Quantitative analysis revealed marked differences in MVD among treatment groups, indicating variable vascular disruption, as summarized in Figure 3A. The control group exhibited the highest vascular density, with a mean vessel count of 11 ± 2 (95% CI: 8 to 14). In contrast, the XRT group showed a notable reduction in vascularity, with a mean of 6 ± 1 (95% CI: 5 to 7), and the MB + FUS group presented a comparable mean vessel count of 6 ± 1 (95% CI: 5 to 7). The MB + FUS + XRT group demonstrated the most pronounced anti-vascular effect, with a mean of only 2 ± 0.4 (95% CI: 2 to 3). Statistical analysis using Brown-Forsythe (F = 42.53, *p* = 0.0002) and Welch’s ANOVA (W = 55.14, *p* < 0.0001) confirmed a statistically significant effect of treatment, even accounting for heterogeneity of variance. Dunnett’s T3 multiple comparisons post hoc test revealed significant reductions in vessel count in the XRT (MD = 5, 95% CI: 1 to 10, *p* = 0.0168), MB + FUS (MD = 5, 95% CI: 1 to 9, *p* = 0.0245), and MB + FUS + XRT (MD = 9, 95% CI: 4 to 13, *p* = 0.0043) groups relative to the control. While no significant difference was observed between XRT and MB + FUS (*p* = 0.9819), the MB + FUS + XRT group exhibited a significantly lower vessel count than either XRT (*p* = 0.0008) or MB + FUS (*p* = 0.0007), respectively.

#### 3.1.6. HIF-1α

HIF-1α, a transcription factor stabilized under hypoxia and linked to angiogenesis and tumour survival, was analyzed to assess treatment-induced hypoxia from vascular disruption by MB + FUS + XRT. The mean percentage of HIF-1α-positive staining was observed as follows: Control (9% ± 3%), MB + FUS (12% ± 2%), XRT (21% ± 4%), and MB + FUS + XRT (22% ± 4%), illustrated in Figure 3C. One-way ANOVA revealed a statistically significant difference in HIF-1α expression among the treatment groups (F = 15.11, *p* < 0.0001). This model accounted for a large proportion of the variance in HIF-1α expression, as demonstrated by a substantial R^2^ value of 0.7640 (76.4%). The Brown-Forsythe test indicated that the assumption of equal variances was met (F = 0.4653, *p* = 0.7111). Post hoc analysis using Tukey’s HSD test, which is appropriate for pairwise comparisons with equal variances, showed that the MB + FUS + XRT group had significantly higher HIF-1α expression compared to the Control group (MD = −19, 95% CI: −28 to −10, *p* < 0.0001), the MB + FUS group (MD = −16, 95% CI: −25 to −7, *p* = 0.0006), and the XRT group (MD = −11, 95% CI: −21 to −0.5, *p* = 0.0392).

#### 3.1.7. VEGF

VEGF, an angiogenic factor regulated by HIF-1α, was assessed to evaluate treatment-induced changes in tumour vascular signalling after MB + FUS + XRT. The mean percentage of VEGF-positive staining was quantified as an indicator of adaptive response to treatment-induced vascular damage. The control group exhibited the lowest mean response at 18% ± 10% (95% CI: 6 to 31). The MB + FUS group showed a mean of 28% ±10% (95% CI: 16 to 41). The combined MB + FUS + XRT group exhibited the second-highest response, with a mean of 33% ± 6% (95% CI: 28 to 39). The XRT group showed the highest response with a mean of 36% ± 4% (95% CI: 30 to 43).

One-way ANOVA revealed a statistically significant difference among group means (F = 4.632, *p* = 0.0174), with treatment accounting for 48% of the total variance (R^2^ = 0.4809). Tests for homogeneity of variance confirmed that variances were not significantly different (Brown-Forsythe: F = 1.341, *p* = 0.2984, Bartlett’s test: χ^2^ = 4.290, *p* = 0.2318). Post hoc analysis using Tukey’s multiple comparisons test revealed that both the XRT (MD = 18, *p* = 0.0198, 95% CI: 3 to 33) and MB + FUS + XRT (MD = 15, *p* = 0.0400, 95% CI: 1 to 30) groups had significantly higher percentages of VEGF-positive staining compared to the control group. The difference between control and MB + FUS (MD = 10, *p* = 0.2128, 95% CI: −4 to 25) was not statistically significant, depicted in Figure 3B. No other pairwise comparisons were statistically significant.

### 3.2. Power Doppler

**Figure 4:**
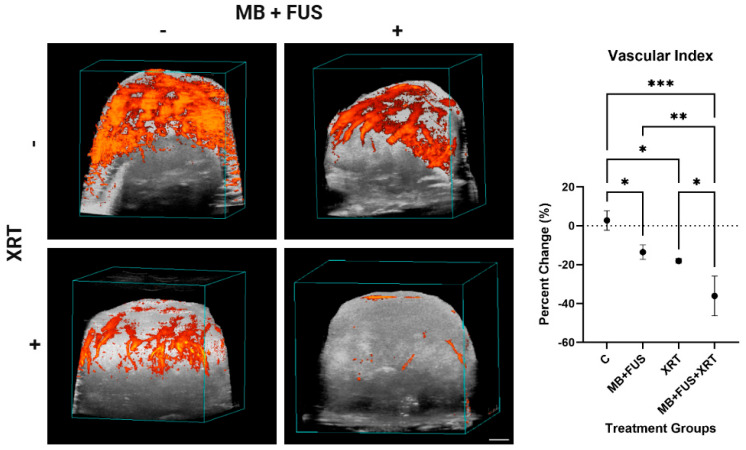
In vivo assessment of tumour perfusion using 3D Power Doppler (PD) ultrasound imaging. Left panel: Representative volumetric reconstructions of tumour vasculature (orange) are shown for each treatment group. Vascularity is visualized in a 3D overlay on B-mode grayscale anatomical images. Scale bar: 2 mm. Right panel: Average Vascular index (VI) across control (C), microbubble-enhanced focused ultrasound (MB + FUS), single-dose radiation therapy (XRT, 8 Gy), and MB + FUS + XRT treatment groups. Data are presented as mean ± SD. *p*-values ranging from *p* < 0.05 (*), *p* < 0.01 (**), *p* < 0.001 (***).

Power Doppler (PD) imaging analysis revealed progressive reduction in vascular signal across treatment groups, quantified as a change in Vascular Index (VI), as presented in Figure 4. The VI was used to assess the impact of treatment on tumour perfusion. The control group exhibited a minimal change (3 ± 5), consistent with physiological variability in baseline vascular signal. In contrast, the MB + FUS group exhibited a moderate reduction in VI (−14 ± 4), while the XRT group showed a slightly greater reduction (−18 ± 1). The MB + FUS + XRT group displayed the largest vascular suppression (−36 ± 10).

One-way ANOVA confirmed a significant effect of treatment on VI (F = 20.36, *p* = 0.0004, R^2^ = 0.8842), indicating that approximately 88% of the variance could be attributed to treatment differences. Assessment of variance homogeneity showed no significant heterogeneity (Brown-Forsythe test: F = 1.227, *p* = 0.3616), supporting the robustness of the ANOVA results. Post hoc analysis using Tukey’s test revealed significant differences between several groups. Specifically, the control group showed significantly higher VI compared to MB + FUS (MD = 16, 95% CI: 1 to 31, *p* = 0.0338), XRT (MD = 21, 95% CI: 3 to 39, *p* = 0.0243), and MB + FUS + XRT groups (MD = 39, 95% CI: 23 to 55, *p* = 0.0003). Additionally, the MB + FUS + XRT cohort exhibited a significantly greater VI reduction compared to the MB + FUS (MD = 23, 95% CI: 8 to 38, *p* = 0.0059) and XRT (MD = 18, 95% CI: 0.1 to 35, *p* = 0.0490) treatments. No other pairwise comparisons were statistically significant.

## 4. Discussion

This study presents compelling histopathological and immunohistochemical evidence supporting the enhanced therapeutic efficacy of combining MB + FUS with XRT. Across multiple biomarkers, including nuclear morphology, apoptosis, proliferation, ASMase-mediated cellular stress, vascular integrity, hypoxia, and angiogenic signalling, the MB + FUS + XRT treatment group consistently demonstrated superior outcomes compared to individual therapies.

The acoustic parameters used in this study (500 kHz, 570 kPa peak negative pressure, 50 ms pulse duration at a 25% duty cycle) were chosen based on previous studies that demonstrated efficient vascular disruption and inertial cavitation without thermal damage [16,26,31]. Unlike many prior studies, which are limited to small animal models or employ less clinically adaptable platforms, this study utilized a 6144-element MRgFUS system with real-time imaging and spatial precision, improving clinical translatability. Moreover, by combining MB + FUS with XRT, we observed synergistic effects that exceeded the impact of USMB or XRT alone, particularly in terms of vascular collapse (Factor VIII loss), apoptosis (TUNEL), and proliferative arrest (Ki-67), offering a significant therapeutic advantage over monotherapy approaches [28,33,36].

A progressive and statistically significant reduction in nuclear size was observed across treatment modalities, with the MB + FUS + XRT combination producing the most pronounced effect (41%) compared to control and individual treatment groups. This finding reinforces the role of nuclear condensation as a marker of apoptosis, consistent with previous reports on the radio-enhancing and cytotoxic effects of MB + FUS and XRT [3,11,38,39]. Notably, while XRT and MB + FUS alone decreased nuclear size by 31% and 17%, respectively, their combination produced a synergistic enhancement. These effects likely stem from MB + FUS-induced vascular and mechanical disruption, which primes tumour cells for subsequent radiation-induced DNA damage and cell death [3,11,29,39,40].

This synergism was further validated by TUNEL staining, where apoptotic indices were markedly higher in the MB + FUS + XRT group (18%) compared to XRT (6%) or MB + FUS (7%) alone. All treatment groups demonstrated a statistically significant increase in TUNEL-positive detection compared to the untreated control (2%), with the MB + FUS + XRT combination exhibiting the most dramatic effect, indicating a synergistic enhancement of DNA fragmentation and cell death [3,36,40,41]. This is consistent with the mechanistic actions of both MB + FUS and XRT, which converge on intrinsic apoptotic pathways [6,33,42]. The amplified TUNEL staining in the combination group supports the hypothesis that MB + FUS acts as a radiation enhancement modality, potentially due to cavitation-induced mechanical disruption of endothelial cells, increased vascular permeability, diminished interstitial pressure, and mechanically induced cell stress [36,39,41,43]. These mechanisms activate ROS production, mitochondrial membrane depolarization, and initiate caspase cascades, all of which promote DNA fragmentation and cell death [26,36,39,43]. Therefore, the synergistic increase in TUNEL-positive cells likely reflects accelerated execution of apoptosis beyond the additive effects of the monotherapies.

Notably, the rapid and extensive apoptosis, as indicated by elevated TUNEL-positive staining, is associated with tumour regression and improved therapeutic response in preclinical oncology studies [3,26,36,41]. However, given that TUNEL staining also detects DNA fragmentation during necrosis and other forms of regulated cell death (e.g., pyroptosis, ferroptosis), additional validation is warranted to distinguish between these pathways accurately [39,44]. In this context, the use of complementary markers, such as cleaved caspase-3, Annexin V, or γ-H2AX, in future studies would help confirm the apoptotic nature of the observed cell death and rule out non-apoptotic or necrotic mechanisms [39,40,44].

ASMase plays a central role in the stress-induced apoptotic cascade, especially in endothelial and tumour cells exposed to mechanical, oxidative, or radiological injury [16,28,30]. Upon activation, ASMase hydrolyzes sphingomyelin into ceramide, a pro-apoptotic lipid messenger that promotes membrane reorganization, mitochondrial permeabilization, and caspase activation, key stages in programmed cell death [16,30,45]. In the context of radiotherapy, ceramide accumulation has been shown to mediate endothelial apoptosis, leading to vascular collapse and impaired perfusion, mechanisms strongly implicated in tumour radio-enhancement [16,28,33,45,46]. The pronounced increase in ASMase expression in the MB + FUS + XRT group corroborates the increased TUNEL staining and vascular disruption initiated by MB + FUS. The upregulation of ASMase across treatment groups further underscores the biological stress induced by MB + FUS and XRT [16,46]. The highest ASMase expression was observed in the MB + FUS + XRT group, suggesting that this enzyme may serve as an early biomarker of treatment response. Given ASMase’s role in ceramide production and apoptosis initiation, this axis warrants deeper investigation using protein quantification techniques (e.g., Western blot, mass spectrometry) and temporal mapping post-treatment [16]. These converging findings suggest that MB + FUS may potentiate radiation-induced vascular damage by promoting ceramide production through ASMase activation, as supported by prior models of ultrasound-enhanced ceramide signalling [16,33,35]. Moreover, ASMase is involved not only in apoptosis but also in membrane permeabilization, inflammatory priming, and autophagy regulation, positioning it as a central regulator of tumour stress responses [16,39]. Its upregulation further supports the rationale for downstream evaluation of ceramide species, caspase activation, and autophagy-related markers (e.g., LC3-II, p62) to delineate the dominant mode of cell death [39,47].

The observed reduction in Ki-67 expression across all treatment groups highlights the anti-proliferative effects of MB + FUS, XRT, and their combination on tumour cell cycling. As a nuclear protein expressed during all active phases of the cell cycle (G1, S, G2, and M), but absent in quiescent (G0) cells, Ki-67 serves as a robust marker of cellular proliferation [4,47,48]. The marked downregulation of Ki-67 in the MB + FUS + XRT group indicates a transition in tumour state toward reduced proliferation and a stressed or apoptotic phenotype following treatment.

This proliferative suppression complements the elevated TUNEL positivity and upregulation of ASMase, both of which indicate enhanced cell death and apoptotic signalling. The drop in Ki-67 may reflect a dual outcome: (1) direct DNA damage and cell cycle arrest induced by XRT, and (2) mechanically mediated microenvironmental stress from MB + FUS, which can impair cellular metabolism, disrupt nutrient delivery, and sensitize cells to apoptosis [1,3,4,26,49]. Together, these treatments appear to shift the tumour population from a proliferative to a terminal fate, as evidenced by histological nuclear changes and magnified cell death. Moreover, under conditions of T-cell immunosuppression (via CsA), the observed Ki-67 suppression further underscores the efficacy of direct cytotoxic and vascular mechanisms, independent of immune-mediated tumour control [48,50]. In immunocompetent settings, T cells can exert indirect anti-proliferative effects through cytokine release (e.g., IFN-γ), immune editing, and pressure on resistant tumour subpopulations [50,51]. The reduction in Ki-67 in this immunosuppressed model indicates that even in the absence of adaptive immune surveillance, MB + FUS + XRT can effectively halt cell cycle progression, likely through tumour-intrinsic and stromal interactions [26,50,51].

Furthermore, decreased Ki-67 expression may also signal loss of angiogenic support, aligning with reduced VEGF expression and diminished vascular integrity. Tumour proliferation is tightly linked to vascular function, and as such, treatment-induced vascular damage, whether via ASMase-mediated pathways or direct endothelial loss, can impair proliferation and promote cell death [3,19]. The overall effects of USMB are initiated primarily at the vascular interface, but our histological analyses (ASMase, TUNEL, Ki-67) demonstrated treatment responses within the tumour parenchyma as well, indicating a secondary propagation of stress and apoptotic signalling beyond the vasculature. This aligns with prior reports that vascular collapse can produce hypoxia, nutrient deprivation, and apoptotic cascades within parenchymal tumour cells [16,26,29]. The larger rabbit model provided an opportunity to observe these effects over a greater spatial scale, reinforcing the translational relevance of MB + FUS + XRT.

Combination therapy significantly disrupted tumour vascular integrity, as evidenced by a reduction in vessel density (Factor VIII staining) and PD imaging analysis. The MB + FUS + XRT group exhibited the lowest vessel density and vascular index (VI = 2), suggesting a synergistic vascular collapse. Mechanistically, MB cavitation may sensitize the tumour endothelium to radiation via ASMase-ceramide signalling, enhanced permeability and vascular disruption [26,28,33,45]. Despite their similar individual effects on vascular metrics, XRT and MB + FUS combined demonstrated synergistic anti-vascular impacts. This suggests that mechanical and radiological strategies can be effectively integrated to target tumour vasculature and improve therapeutic outcomes while minimizing systemic toxicity.

The analysis of Factor VIII staining provides direct histological evidence of treatment-induced endothelial disruption and microvessel loss [52]. Diminished Factor VIII expression reflects the structural degradation of intratumoral vasculature, most prominently in the MB + FUS + XRT group, where staining was substantially reduced. In our study, all treatment groups showed reduced Factor VIII staining compared to controls, with the most pronounced loss observed in the MB + FUS + XRT combination group, indicating a significant compromise in vascular architecture. This supports a model in which combination therapy produces a synergistic anti-vascular effect, driven by both radiation-induced apoptosis and MB + FUS-mediated mechanical injury [52].

These histological findings are congruent with the PD imaging data, which revealed a substantial decrease in functional blood flow in treated tumours, exhibited most prominently in the MB + FUS + XRT cohort. The alignment between decreased PD signal and reduced Factor VIII staining suggests that the observed flow loss is not solely due to vasoconstriction or transient hemodynamic changes, but rather to permanent vascular damage and endothelial cell loss. The integration of structural histology and functional imaging supports the interpretation that combination therapy mediates vascular disruption, contributing to nutrient deprivation, hypoxia, and downstream tumour cell death [31,52]. Furthermore, the convergence of these vascular changes with increased ASMase expression, TUNEL positivity, and Ki-67 suppression reinforces a mechanistic link between vascular injury, apoptosis induction, and proliferative arrest. In the xenograft model used here, where immune-driven anti-angiogenic mechanisms are largely inactive, the observed vascular collapse, evidenced by reduced Factor VIII and PD signal attenuation, likely results from direct endothelial injury induced by MB + FUS and XRT. These effects may be further exacerbated by ceramide accumulation resulting from ASMase activation, as ceramide signalling is known to induce endothelial apoptosis and impair vessel stability [33,36,52]. Together, the integration of PD imaging and Factor VIII histology provides a compelling, multi-modal depiction of treatment-induced vascular disruption, highlighting both real-time perfusion deficits and permanent microvascular collapse as key contributors to therapeutic efficacy. HIF-1α and VEGF staining indicated that vascular damage responses were as expected.

While the primary focus of this study was on treatment response, we did observe regional heterogeneity in perfusion, vascular density, and hypoxia across tumour sections, consistent with the expected complexity of larger xenografts. These findings support the use of the rabbit model to better recapitulate the spatial variability seen in human tumours. However, we did not systematically stratify analysis by tumour subregions (e.g., hypoxic vs. normoxic compartments), and this represents an area for further study in evaluating whether heterogeneous areas differentially respond to MB + FUS + XRT. Additionally, a limitation of this study is the absence of longitudinal efficacy endpoints (e.g., tumour growth delay or survival). Our focus was on acute vascular and cellular responses, given the novelty of scaling MB + FUS + XRT to a larger, clinically relevant model. While our results confirm robust vascular collapse, apoptosis, and proliferative suppression, it remains to be determined whether these acute responses translate to durable tumour control in larger, heterogeneous tumours. Future studies will be needed to evaluate long-term efficacy, particularly in immunocompetent or humanized models, where adaptive immune responses may further influence outcomes.

## 5. Conclusion

In summary, this study provides compelling evidence that the combination of MB + FUS and XRT produces robust antitumour effects in a large-scale rabbit xenograft model. By simultaneously disrupting vascular integrity, initiating endothelial stress, and inhibiting tumour proliferation, the therapeutic combination of MB + FUS + XRT exhibited pronounced effectiveness. Utilizing multiple assessment modalities, including PD-based perfusion imaging, Factor VIII endothelial staining, and nuclear morphometry, revealed significant vascular disruption and nuclear condensation, most prominently in the MB + FUS + XRT treatment group. The notable reduction in nuclear size, a surrogate marker of apoptosis, was corroborated by increased TUNEL positivity and elevated ASMase expression, suggesting that ASMase/ceramide-mediated apoptosis and vascular disruption synergistically contribute to tumour cell death. Additionally, widespread Ki-67 suppression across treated tumours indicates sustained proliferative arrest, further supporting the coordinated cytotoxic and anti-vascular mechanisms driving the therapeutic response.

As a clinically scalable, image-guided system, the FUS system used here permitted precise spatial targeting and consistent MB activation, resulting in radiation enhancement and reproducible vascular disruption across larger tumour volumes. Future investigations should extend this approach to immune-competent or humanized models to capture host-to-tumour interactions, as well as assess long-term control dynamics. Additionally, incorporating functional biomarkers, such as cytokine panels, immune cell profiling, and dynamic imaging of tumour oxygenation and perfusion, will be critical for distinguishing direct treatment effects from host-mediated responses. These efforts will support the rational development of next-generation combination therapies that integrate vascular modulation, radiotherapy, and immunotherapy to achieve durable and clinically applicable cancer treatment.

## Data Availability

The raw data for this project can be made available by the authors upon request.

## Abbreviations

The following abbreviations are used in this manuscript:

USMBs	Ultrasound-stimulated microbubbles
XRT	Radiation Therapy
MRgFUS	MRI-guided focused ultrasound
ROS	Reactive oxygen species
SSBs	Single-strand breaks
DSBs	Double-strand breaks
MBs	Microbubbles
FUS	Focused ultrasound
BBB	Blood–brain barrier
ASMase	Acid sphingomyelinase
DISC	Death-inducing signalling complex
MOMP	Mitochondrial outer membrane permeabilization
PD	Power Doppler
IV	Intravenous
CsA	Cyclosporine A
ROI	Region of interest
VI	Vascular Index
H&E	Hematoxylin and Eosin
TUNEL	Terminal deoxynucleotidyl transferase dUTP nick end labelling assay
HIF-1α	Hypoxia-Inducible Factor 1-alpha
VEGF	Vascular Endothelial Growth Factor
WSI	Whole slide image
